# The accuracy of artificial intelligence in 3D preoperative planning for total hip arthroplasty: A systematic review and meta‐analysis

**DOI:** 10.1002/jeo2.70427

**Published:** 2026-02-19

**Authors:** Seif B. Altahtamouni, Loay A. Salman, Abdallah Al‐Ani, Ghalib Ahmed

**Affiliations:** ^1^ Department of Orthopedic Surgery Hamad General Hospital, Hamad Medical Corporation Doha Qatar; ^2^ Office of Scientific Research and Affairs King Hussein Cancer Center Amman Jordan

**Keywords:** 3D preoperative planning, artificial intelligence, implant sizing, surgical accuracy, total hip arthroplasty

## Abstract

**Purpose:**

This systematic review and meta‐analysis compare AI‐assisted 3‐dimensional (3D) preoperative planning in total hip arthroplasty (THA) to traditional 2‐dimensional (2D) templating.

**Methods:**

PubMed, Scopus, and Embase were searched from inception until October 2024 for studies on the accuracy of 3D preoperative planning in THA. Statistical analysis was performed using R (v4.3.3) with a random‐effects model due to high heterogeneity. Odds ratios with 95% confidence intervals were calculated for dichotomous outcomes. Heterogeneity was assessed using the *I*² statistic, and publication bias was evaluated through funnel plots and Egger's test. The primary outcome was the accuracy of detecting acetabular cup and femoral stem size. This meta‐analysis followed PRISMA guidelines for systematic reviews.

**Results:**

Eight studies with 1371 participants from China were analysed. The mean age was 54.48 ± 12.98 years, and the mean BMI was 24.63 ± 3.73 kg/m². The Newcastle–Ottawa Scale (NOS) scores ranged from 6 to 9. The AI model effectively predicted acetabular cup and femoral stem sizes, with an odds ratio (OR) of 3.85 for the exact cup size (95% CI: 2.79–5.32; *p* < 0.0001) and an OR of 3.49 for predictions within one standard deviation (95% CI: 1.21–10.13; *p* = 0.0212). Heterogeneity was 42% and 81%, respectively. For the femoral stem, the AI achieved an OR of 3.28 for exact size predictions (95% CI: 2.56–4.22; *p* < 0.0001) and an OR of 5.35 for size within one standard deviation (95% CI: 3.84–7.45; *p* < 0.0001), with no significant heterogeneity (*I*² = 0%).

**Conclusion:**

This meta‐analysis confirms that AI‐assisted 3D preoperative planning in THA provides better accuracy for predicting the acetabular cup and femoral stem sizes than traditional 2D templating methods. Further studies with larger sample sizes and more extended follow‐up periods across multiple countries are warranted to validate our findings.

**Level of Evidence:**

Level III.

**Abbreviations**
AI: artificial intelligence
BMI: body mass index
CI: confidence interval
CT: computed tomography
DDH: developmental dysplasia of the hip
NOS: Newcastle–Ottawa Scale
OR: odds ratio
PRISMA: Preferred Reporting Items for Systematic Reviews and Meta‐Analyses
RA: rheumatoid arthritis
RCT: randomised controlled trial
THA: total hip arthroplasty

## INTRODUCTION

Total hip arthroplasty (THA) is an effective surgical intervention for debilitating hip joint disease, such as osteoarthritis, that significantly worsens an individual's quality of life. Successful THA relies on meticulous preoperative planning, surgical technique, and correct positioning of prosthetic implants. Traditionally, two‐dimensional (2D) modalities have been used for planning; however, they have a limitation in accurately representing the complex three‐dimensional (3D) anatomy of the hip joint. Inadequacies in 2D modalities can lead to poor implant positioning, thereby jeopardising implant survival and surgical success [4].

Recent advancements have introduced artificial intelligence (AI) into orthopedic planning. AI‐integrated 3D systems, particularly those based on machine learning and deep learning, utilise patient‐specific anatomical data from CT imaging to predict optimal implant sizes and positions. These systems aim to improve accuracy, reduce planning time, and enhance surgical workflow efficiency [23, 24, 26].

AI‐facilitated 3D preoperative planning software can utilise 3D imaging information to calculate and predict implant size and position with high accuracy. It can attempt to correct mechanical positioning, and AI‐facilitated 3D planning can utilise patient‐specific anatomical information, potentially minimising dislocations and wear and improving function [20]. AI algorithms can even detect minor anatomical information that is not observable in routine imaging, thus enabling a more intelligent and wiser surgical decision [8].

Integrating AI with 3D modalities can even make 3D planning much shorter, and in a shorter duration, surgical workflows can become much more efficient and less costly [15]. Studies have even determined that AI‐powered models can learn from massive datasets and, in the process, gain accuracy and reliability in implant positioning over time [29]. AI‐powered software with augmented visualisation and prediction has been shown to have the potential to provide postoperative improvements, including shorter recovery times and enhanced joint function [7, 34]. Moreover, emerging concepts like digital twins—real‐time, data‐driven virtual representations of patient anatomy—are set to improve surgical planning by using AI and deep learning to simulate outcomes and optimise strategies [9].

Therefore, this meta‐analysis aims to compare the accuracy of AI‐assisted 3D preoperative planning with traditional 2D templating for predicting acetabular and femoral component sizes in THA. By synthesising recent evidence, we aim to assess whether AI enhances implant selection precision.

## METHODS

This systematic review and meta‐analysis was conducted according to the preferred reporting items for systematic reviews and meta‐analyses (PRISMA) [17]. The completed PRISMA checklist is provided as Supporting Information: Material 1. The protocol of this review was registered on the International Prospective Register of Systematic Reviews (PROSPERO).

### Search strategy

A comprehensive search was done through PubMed, Scopus, and Embase. The search was conducted from inception until October 2024 to identify all the studies that examine the use of AI 3D preoperative planning in total hip arthroplasty. A combination of keywords and medical subject headings were used in the search process, including artificial intelligence preoperative planning, AI 3D preoperative planning, AI HIP, total hip arthroplasty and total hip replacement.

### Eligibility criteria

Articles included in this review were prospective and retrospective studies. Studies were deemed eligible if they met the following criteria: (1) studies written in the English language, (2) reporting the accuracy of AI HIP system in detecting acetabular cup and femoral stem sizes, (3) comparing AI HIP system using computed tomography (CT) scans with traditional 2‐D planning, (4) all types of total hip arthroplasty regardless of the preoperative diagnosis, the approach, the use of cemented vs cementless, or the manufacturer of components. Studies were excluded if they: (1) failed to report the accuracy of acetabular and femoral component sizes, (2) did not include a comparison group, (3) studies with incomplete data set and (4) review articles, cadaveric studies and case reports.

### Study screening

Two authors independently reviewed the titles and abstracts to identify relevant articles. Potentially relevant articles were then subjected to full‐text screening for articles that met the pre‐specified eligibility criteria. A third more senior author resolved any disagreement between the two authors.

### Data extraction

Two authors independently extracted data on various aspects of the study, including patient demographics (age, gender and body mass index), preoperative diagnoses, the Dorr classification, the AI model used, the sequence of preoperative planning, the manufacturer, the exact size, the accurate size (within ±1), and the overall accuracy of the acetabular and/or femoral components. The primary outcome measured was the accuracy of component sizes within ±1 size of the AI‐based 3D preoperative planning compared to traditional manual 2D planning.

### Quality appraisal

Two independent authors assessed quality using the Newcastle–Ottawa Scale (NOS) [28]. The NOS is a tool used to assess the quality of non‐randomised studies, especially cohort and case‐control research. It scores studies on three key areas: Selection (how well participants are chosen), Comparability (whether key factors are accounted for), and Outcome (how effectively results are measured). A higher score (out of 9) indicates a stronger study design and a lower risk of bias.

### Statistical analysis

We conducted a meta‐analysis of eligible studies using R (version 4.3.3, R Core Team, Vienna, Austria, 2020) using the meta package (i.e., forest and metabin). Odds ratios (OR) and their associated 95% confidence intervals were expressed for dichotomous variables (e.g., rate of accurate predictions). Heterogeneity among effect sizes was evaluated using the I squared statistic. Definitions for heterogeneity were adapted from the Cochrane Handbook (>25% mild, 25%–50% moderate, >50% severe). Due to the high heterogeneity for the dichotomous variables, a random‐effects model using the DerSimonian–Laird method was applied to account for between‐study variance, as recommended for meta‐analyses with heterogeneous clinical data [22]. Both a funnel plot and Egger's test of asymmetry were utilised to assess publication bias. Visual inspection of funnel plots showed no significant asymmetry, and Egger's test did not indicate publication bias for any of the primary outcomes (*p* > 0.05).

## RESULTS

**Figure 1:**
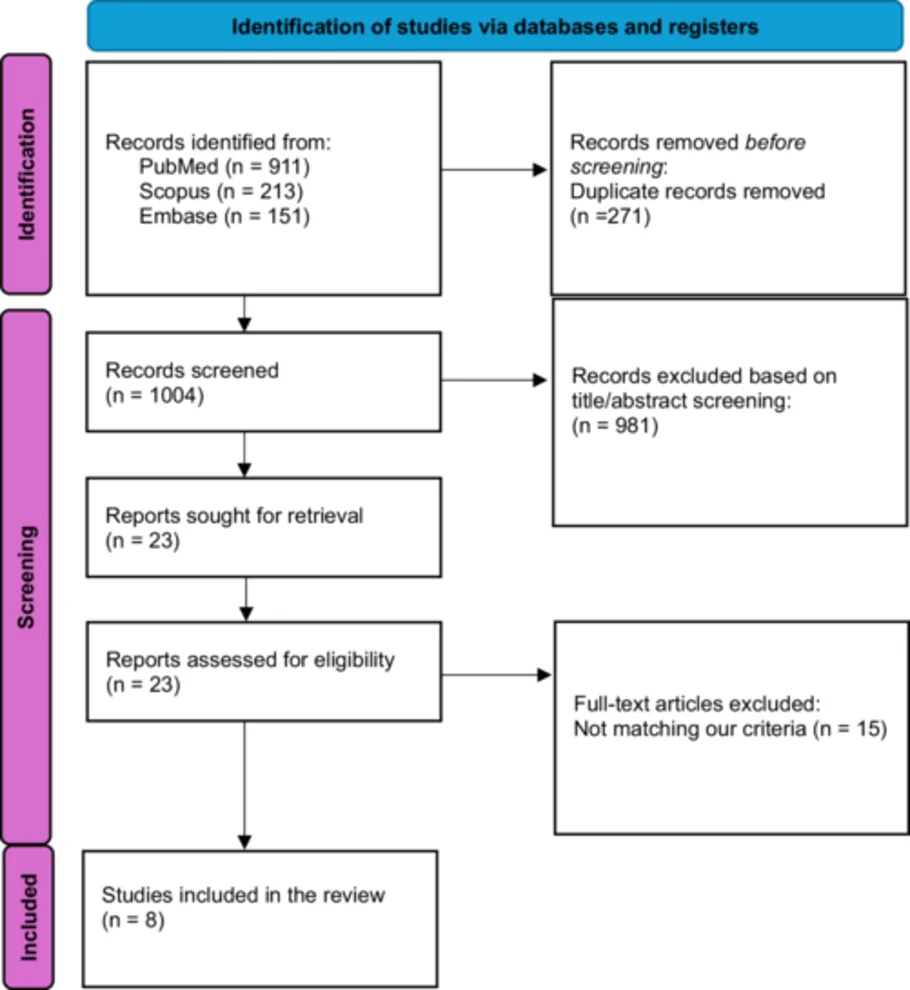
PRISMA flow diagram.

A total of eight studies were included in the final analysis (Figure 1). All studies originated in China. About 38% of studies were prospective cohorts with level II evidence. The remainder of the studies are retrospective with level III evidence. Across the 8 studies, 1371 cases were included, of which 49% were males and 51% were females. The mean pooled age and BMI for the included cases were 54.48 ± 12.98 years and 24.63 ± 3.73 kg/m^2^, respectively. A summary of the characteristics of the articles included is presented in Table 1.

**Table 1:** Characteristics of included studies.

Study	Country	Design, LoE	Period	Imaging modality		No. of patients			Gender (M/F)	Age (Mean ± SD)	BMI (Mean ± SD)
				AI HIP	2D planning	AI HIP	2D planning	Total			
Anwar et al. [2]	China	Prospective, II	Nov 2020– May 2021	CT‐scan	X‐rays	117	117	117	52/65	62.3 ± 10.7	25.5 ± 3.4
Chen et al. [6]	China	Prospective, II	Oct 2019–Feb 2021	CT‐scan	X‐rays	60	60	120	AI HIP: 29/31 2D planning: 32/28	AI HIP: 47.62 ± 15.3 2D planning: 53.75 ± 16.1	AI HIP: 24.19 ± 3.08 2D planning: 25.14 ± 3.78
Ding et al. [10]	China	Retrospective, III	Apr 2019–Jun 2020	CT‐scan	X‐rays	316	316	316	192/124	50.68 ± 12.64	25.07 ± 3.2
Huo et al. [12]	China	Prospective, II	Oct 2019–Jul 2020	CT‐scan	X‐rays	53	53	53	29/24	57.4 ± 1.7	NS
Wu et al. [31]	China	Retrospective, III	Jan 2020–July 2022	CT‐scan	X‐rays	95	66	161	AI HIP: 57/38 2D planning: 44/22	AI HIP: 57.5 ± 10.5 2D planning: 57.8 ± 10.6	AI HIP: 25.3 ± 3.0 2D planning: 24.7 ± 3.8
Wu et al. [30]	China	Retrospective, III	Jan 2020– July 2022	CT‐scan	X‐rays	34	27	61	AI HIP: 16/18 2D planning: 12/15	AI HIP: 58.2 ± 9.8 2D planning: 60.5 ± 11.1	AI HIP: 25.9 ± 2.6 2D planning: 26.1 ± 3.8
Xie et al. [32]	China	Retrospective, III	May 2019–Aug 2023	CT‐scan	X‐rays	103	103	103	16/87	42.3 ± 12.4	24.0 ± 2.7
Yang et al. [33]	China	Retrospective, III	Jun 2019–Mar 2022	CT‐scan	X‐rays	220	220	440	AI HIP: 92/128 2D planning: 101/119	AI HIP: 56.6 ± 14.5 2D planning: 57.4 ± 15.2	23.61
Abbreviations: 3D, three‐dimensional; AI, artificial intelligence; CT, computed tomography; LoE, level of evidence; NS, not stated; SD, standard deviation.											

Study	No. Hips	Surgical approach	Preoperative diagnoses						Dorr classification			AI Model	Manufacturer	
			Osteonecrosis of the femoral head	Osteoarthritis	Femoral neck fracture	Ankylosing spondylitis	RA	DDH	A	B	C		Acetabular Cup	Femoral stem
Anwar et al. [2]	117	Posterolateral	39	25	25	0	0	28	15	90	12	AI HIP	Pinnacle (DePuy)	Corail, Summit or Trilock, (DePuy)
Chen et al. [6]	120	Posterior	74	10	3	11	1	21	NA	NA	NA	AI HIP	Pinnacle (DePuy)	Corail, or Trilock (DePuy)
Ding et al. [10]	316	Posterolateral	234	10	7	7	8	50	24	206	86	AI HIP	Pinnacle (DePuy)	Trilock (DePuy)
Huo et al. [12]	59	Posterolateral	16	16	0	9	2	16	NA	NA	NA	AI HIP	Pinnacle (DePuy)	Summit or Corail (DePuy)
Wu et al. [31]	161	Posterolateral	68	23	3	2	5	60	24	137	0	AI HIP	Pinnacle (Johnson & Johnson)	Summit (Johnson & Johnson)
Wu et al. [30]	61	Posterolateral	61	0	0	0	0	0	9	52	0	AI HIP	Pinnacle (Johnson & Johnson)	Summit (Johnson & Johnson)
Xie et al. [32]	164	Posterolateral	0	0	0	0	0	164	NA	NA	NA	AI HIP	Pinnacle (DePuy)	NS
Yang et al. [33]	440	Direct anterior	177	80	103	0	0	80	NA	NA	NA	AI HIP	Pinnacle (DePuy)	Trilock (DePuy)

### Quality assessment

Most of the studies scored well, ranging from 7 to 9 out of 9, reflecting strong research design and clear methodology. Wu 2023 (2) and Yang 2023 (9/9) demonstrated excellent study quality, while Ding 2021 (6/9) had some limitations in exposure assessment and follow‐up. Overall, the studies were robust, but findings from lower‐scoring ones should be interpreted cautiously. A summary of the quality assessment is presented in Table 2.

**Table 2:** Quality assessment using the Newcastle–Ottawa Scale (NOS).

Study	Selection				Comparability	Outcome			Total Score (out of 9)
	Representativeness of the Exposed Cohort	Selection of the Non‐Exposed Cohort	Ascertainment of Exposure	Demonstration that Outcome of interest Was Not Present at Start of study	Comparability of cohorts based on design or analysis	Assessment of Outcome	Was Follow‐up Long Enough for Outcomes to Occur?	Adequacy of Follow‐up of Cohorts	
Anwar et al. [2]	1	1	1	1	1	1	1	0	7
Chen et al. [6]	1	1	1	1	2	1	1	0	8
Ding et al. [10]	1	1	0	1	1	1	1	0	6
Huo et al. [12]	1	1	1	1	1	1	1	1	8
Wu et al. [31]	1	1	1	1	2	1	1	0	8
Wu et al. [30]	1	1	1	1	2	1	1	1	9
Xie et al. [32]	1	1	0	1	1	1	1	1	7
Yang et al. [33]	1	1	1	1	2	1	1	1	9

### Acetabular cup

**Figure 2:**
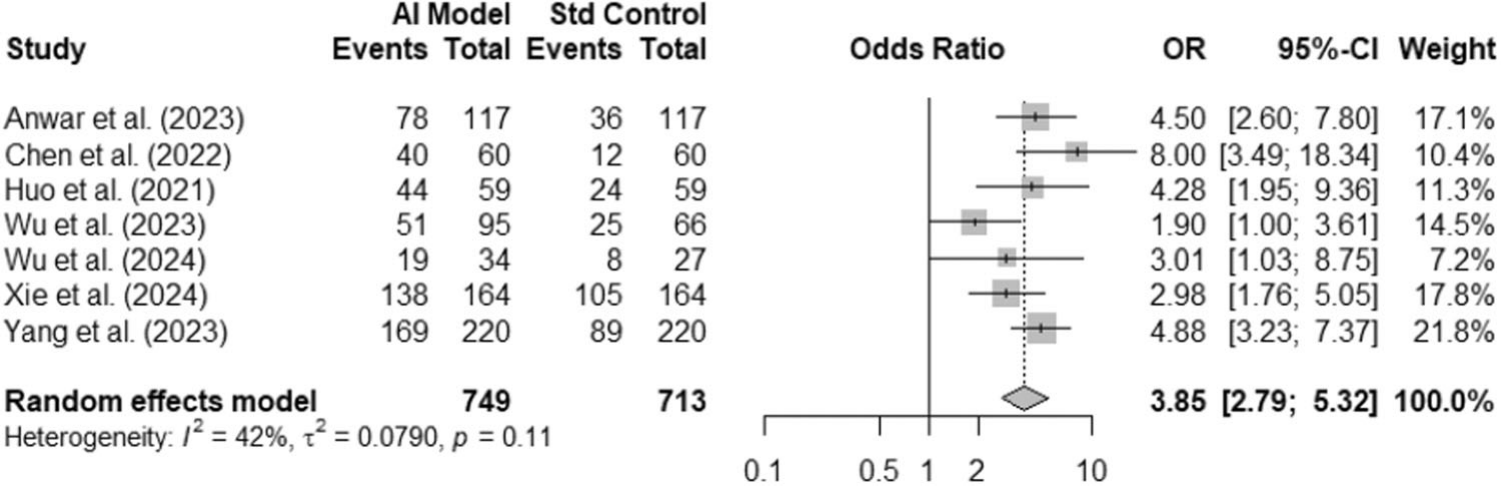
Forest plot of acetabular cup exact size prediction. AI, artificial intelligence; CI, confidence interval; OR, odds ratio.

**Figure 3:**
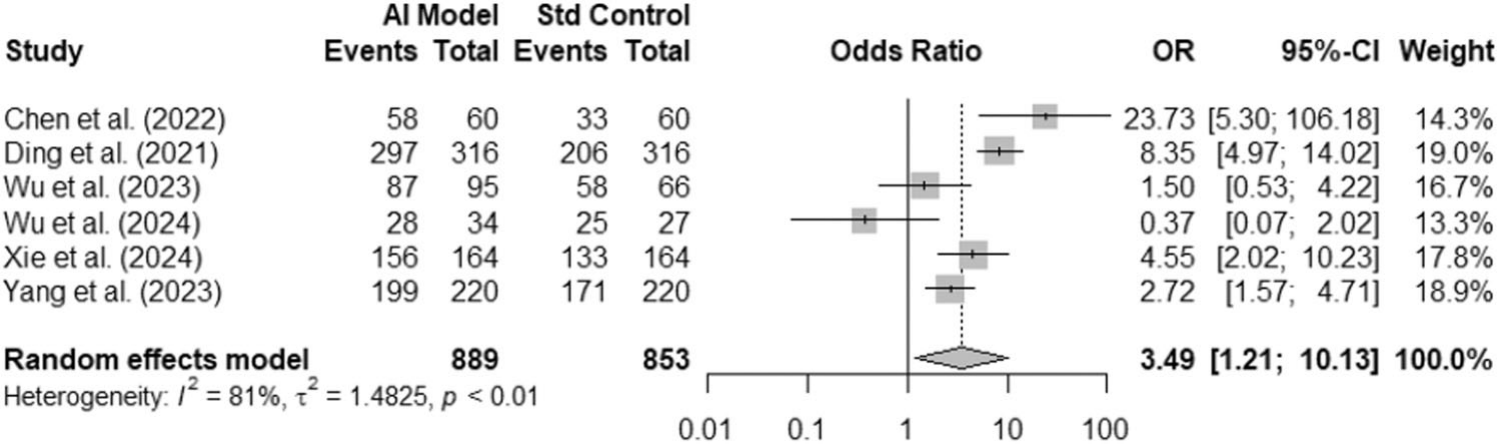
Forest plot of acetabular cup size prediction within one standard deviation. AI, artificial intelligence; CI, confidence interval; OR, odds ratio.

Among seven studies with 1462 observations, the AI model was significantly more likely to predict the exact size of the acetabular cup (OR: 3.85; 95%CI: 2.79–5.32; *p*: <0.0001) (Figure 2). Heterogeneity was insignificant at an *I* ^2^ of 42%. Similarly, across six studies with 1742 observations, the AI model was significantly more likely to predict the size of the acetabular cup size within 1 standard deviation (OR: 3.49; 95%CI: 1.21–10.13; *p*: 0.0212) (Figure 3). There was significant heterogeneity for the aforementioned OR at an *I* ^2^ of 81%.

### Femoral stem

**Figure 4:**
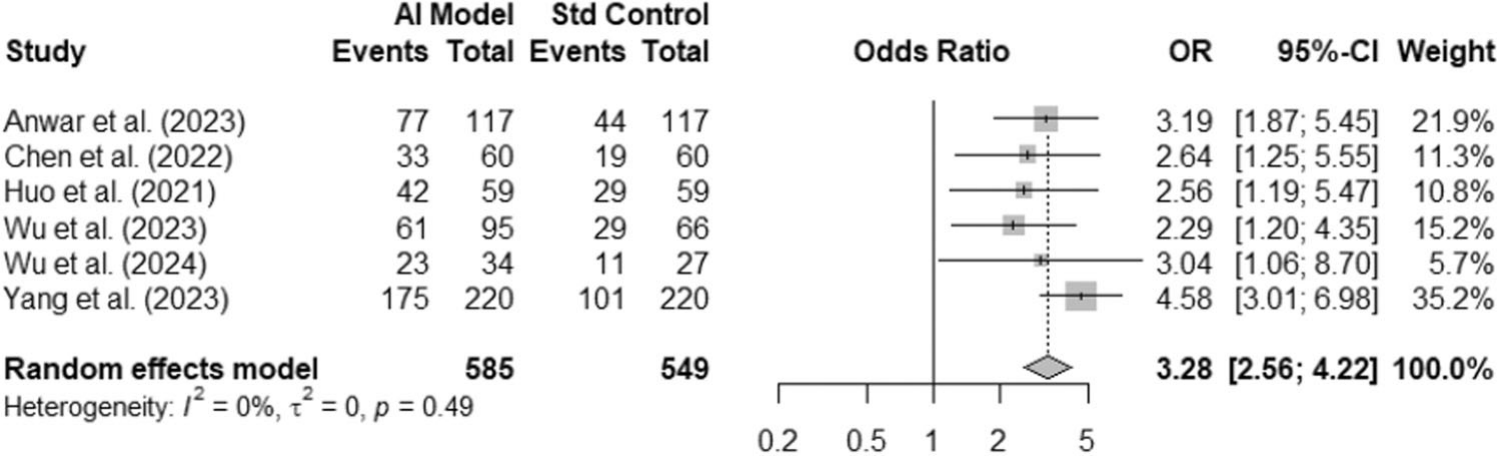
Forest plot of femoral stem exact size prediction. AI, artificial intelligence; CI, confidence interval; OR, odds ratio.

**Figure 5:**
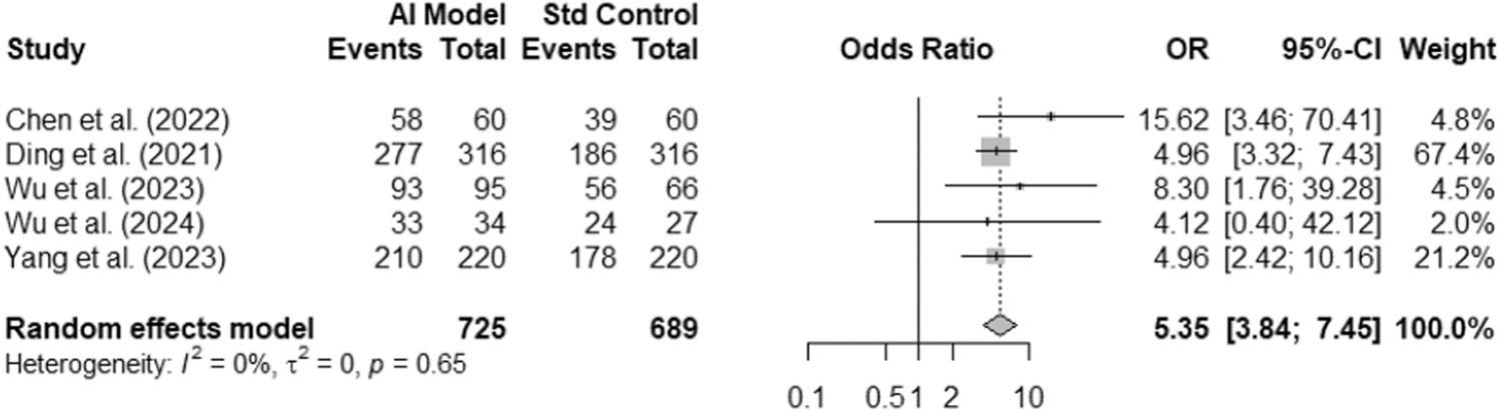
Forest plot of femoral stem size prediction within one standard deviation. AI, artificial intelligence; CI, confidence interval; OR, odds ratio.

Across six studies with 1134 observations, the exact size of the femoral stem was significantly predicted more accurately by the AI model compared to the standard control (OR: 3.28; 95%CI: 2.56–4.22; *p*: <0.0001) (Figure 4). Similarly, across five studies with 1414 observations, the AI model was significantly more likely to predict the size of the femoral stem within 1 standard deviation (OR: 5.35; 95%CI: 3.84–7.45; *p*: <0.0001) (Figure 5). Heterogeneity was insignificant at an *I* ^2^ of 0% for both associations.

## DISCUSSION

This systematic review and meta‐analysis suggest that artificial intelligence 3‐dimensional preoperative planning in THA improves both stem and cup size selection, enhancing surgical efficacy and improving postoperative outcomes. Mozafari et al. [18] conducted a systematic review that reported similar outcomes. It included six studies and found that AI‐assisted planning yielded better outcomes than 2D traditional templating regarding femoral and acetabular positioning.

Our meta‐analysis showcases the advantages of AI‐driven 3D planning over traditional templating and provides quantitative data to support the findings. Our results demonstrated a significantly higher accuracy in predicting the exact size and/or within one standard deviation for the acetabular cup and femoral stem sizes. These findings were consistent with the current literature. For instance, Anwar et al. [2] reported a 66.7% accuracy for cup sizes using AI planning, which was significantly higher than the reported accuracy of traditional planning. Additionally, AI HIP software reached 94% and 87.7% agreement for both cup size and stem size, respectively, as reported by Ding et al. [10]. Hou et al. [12] found that AI‐driven 3D planning had 74.58% accuracy for acetabular cups and 71.19% for femoral stem sizes. Wu et al. [30] found that AI‐assisted planning placed 91.2% of acetabular cups within the Lewinnek safe zone, indicating a more precise placement. Another study by Wu et al. [31] reported 54% and 64% accuracy rates for acetabular and femoral prostheses, respectively. Moreover, AI HIP software reached an 84.1% accuracy rate within ±0 size for predicting acetabular cup size, as reported by Xie et al. [32]. Finally, Yang et al. [33] showed a higher accuracy of AI HIP in predicting acetabular cup (76.8%) and femoral stem (79.5%), which was significantly higher than what manual 2D templating predicted.

AI has recently been widely used in orthopedic procedures. For example, pedicle screw placements in spine surgeries, knee replacement, and shoulder arthroplasty procedures have recently incorporated the use of AI to reach better implant alignment and precise component positioning [13, 14, 25]. Beyond orthopaedics, AI has also made its way into robotic‐assisted and craniofacial reconstructive surgeries [5], showing its growing influence in medicine. It has also been explored in joint replacement planning through predictive modelling and workflow optimisation [11, 27].

Despite widely used traditional templating methods, it has multiple challenges, such as predicting accurate and unique anatomical differences [21]. AI can overcome these challenges by using machine learning models to analyse CT scan data and generate precise predictions regarding implant dimensions and placements [6]. These advantages increase AI's ability to improve implant size predictions, leading to better outcomes [10].

The reliability and practicality of deploying AI into real‐world orthopedic workflows are further emphasised by growing literature on clinical utility and performance benchmarking in preoperative planning [16, 25]. Studies have shown that AI‐supported planning reduces preoperative preparation time and decreases the need for intraoperative fluoroscopy [33]. In one study, Chen et al. [6] reported that their AI‐based system completed preoperative planning in just 1.86 min per case, compared to 185.4 minutes required for manual planning, further emphasising AI's ability to reduce intraoperative workload and support faster decision‐making. Given that longer surgeries are associated with higher risks of infection, bleeding, and perioperative complications [1], AI's potential to streamline procedures may lead to real‐world clinical benefits. Beyond efficiency, AI improves implant positioning, which is critical for long‐term success in THA. Poorly positioned implants can increase the risk of instability, impingement, and accelerated wear [3].

Despite all the advantages of AI‐assisted planning, multiple challenges, including cost, accessibility, and surgeon acceptance, remain key barriers to widespread adoption [15]. Moreover, consistency of the results across studies can arise due to differences in AI software and training datasets [29]. Ethical challenges, including algorithmic bias, patient data privacy, and the interpretability of results, are also of concern [19]. These challenges prompt the need for more validation through large‐scale clinical trials across multiple worldwide institutions [7, 34]. Further research should focus on standardising AI models and exploring their integrations with robotic‐assisted surgeries.

### Study limitations

While our systematic review highlights the benefits of AI‐assisted preoperative planning, it also has certain limitations.

#### Geographic limitation

All included studies were conducted in China, raising concerns about whether AI‐assisted planning would perform similarly across different patient populations.

#### Study design and bias

All included studies were retrospective or prospective, making them susceptible to bias. Randomised controlled trials (RCTs) are necessary for more substantial, more conclusive evidence.

#### Variability in 2D planning techniques

The studies in our review used different 2D planning methods, leading to methodology inconsistencies.

#### Lack of long‐term data

Many studies only had short follow‐up periods (under two years), making it difficult to assess the long‐term durability of AI‐planned THA.

## CONCLUSION

AI‐assisted 3D preoperative planning significantly improves implant size prediction accuracy for both acetabular and femoral components compared to traditional 2D templating. While our findings support the growing role of AI in surgical planning, we did not directly assess clinical outcomes or intraoperative efficiency. Future large‐scale, multicenter studies should evaluate the clinical impact of AI integration, including operative time, complication rates, and long‐term implant survival.

## AUTHOR CONTRIBUTIONS

All authors contributed to the study's conception and design. Seif B. Altahtamouni and Loay A. Salman performed material preparation, literature review, data collection, and quality assessment. Abdallah Al‐Ani performed statistical analysis. Seif B. Altahtamouni wrote the first draft of the manuscript, and all authors commented on previous versions. All authors read and approved the final manuscript.

## CONFLICT OF INTEREST STATEMENT

The authors declare no conflicts of interest.

## ETHICS STATEMENT

PROSPERO: CRD42024606670.

## Supporting information

Supporting information.

## Data Availability

Available upon request.
